# Container-based framework for large-scale spiking network simulation

**DOI:** 10.3389/fnins.2026.1893064

**Published:** 2026-09-09

**Authors:** Oliver James, Sungho Hong

**Affiliations:** Center for Memory and Glioscience, Institute for Basic Science, Daejeon, Republic of Korea

**Keywords:** graphics processing unit (GPU), high-performance computing (HPC), large-scale simulation, reproducibility, spiking neural network

## Abstract

High-performance computing (HPC) is critical for simulating large-scale neural networks with detailed biophysics, yet deploying these simulations across heterogeneous computing infrastructures remains a significant challenge due to software dependencies and hardware variability. This study introduces a framework utilizing containerization technology to ensure scalable and reproducible simulations by encapsulating the entire software stack, including compilers, MPI libraries, and GPU toolchains, within a portable image. We created a spiking network model with millions of neurons and synapses by replicating the cerebellar neural circuit module and performed benchmarking with it. Our results demonstrate that containerized simulations introduce little performance penalty compared to native installations while delivering identical results across multiple systems with minimal effort, providing a robust solution for portability. This framework can accelerate the scalable development of spiking network models, facilitate the precise reproduction of complex workflows and simplify collaboration, addressing major barriers in modern computational neuroscience research.

## 1 Introduction

Computational modeling has become an essential tool for understanding the structure and dynamics of neural systems. Since the pioneering work on mathematical descriptions of neuronal excitability and population dynamics (Hodgkin and Huxley, 1952; Wilson and Cowan, 1972), computational approaches have played a central role in linking experimental observations to theoretical frameworks. Over the past decades, advances in computational neuroscience have enabled increasingly sophisticated models that capture neuronal activity across multiple spatial and temporal scales (Dayan and Abbott, 2001; Gerstner et al., 2014; Koch and Segev, 1998; Sterratt et al., 2011). At the same time, progress in experimental techniques has produced increasingly detailed datasets describing neuronal morphology, synaptic connectivity, and electrophysiological properties. These developments have enabled the construction of biologically realistic models that attempt to reproduce the behavior of neural circuits at both cellular and network levels (Brette et al., 2007). However, the growing complexity of such models often leads to substantial computational demands, making high-performance computing (HPC) an essential component of modern computational neuroscience research (Arkhipov et al., 2025; Brette et al., 2007; D’Angelo et al., 2013).

Large-scale neural simulations frequently involve millions of neurons and billions of synaptic interactions, requiring distributed computing architectures and efficient numerical solvers. To support such simulations, a variety of neural simulation platforms have been developed, including NEST, Brian, and NEURON (Gewaltig and Diesmann, 2007; Goodman and Brette, 2009; Hines and Carnevale, 1997). Among these, the NEURON simulation environment has become widely adopted for modeling biophysically detailed neurons and networks (Hines and Carnevale, 1997). More recently, optimized computational engines such as CoreNEURON have been introduced to improve the performance and scalability of large-scale simulations by exploiting distributed parallelism and hardware accelerators such as graphics processing units (GPUs) (Kumbhar et al., 2019). These developments have enabled increasingly large and detailed neural circuit simulations (Billeh et al., 2020; Dura-Bernal et al., 2023; Potjans and Diesmann, 2014; Hjorth et al., 2020; Markram et al., 2015; Sudhakar et al., 2017; Yamaura et al., 2020).

Despite these advances, deploying large-scale neural simulations across diverse HPC infrastructures remains challenging. HPC systems differ widely across institutions in terms of operating systems, compiler toolchains, message-passing libraries, GPU drivers, and runtime dependencies. Consequently, installing and maintaining complex scientific software environments can be time-consuming and error prone. These difficulties are further amplified when simulation workflows must be migrated between computing clusters or shared among collaborators. Such issues present a significant barrier to reproducibility, which has become a major concern in computational science (Stodden et al., 2016).

Containerization technologies have recently emerged as a promising solution to these challenges by providing portable and reproducible software environments (Boettiger, 2015; Kurtzer et al., 2017; Merkel, 2014). Containers package applications together with all required software dependencies while maintaining lightweight isolation from the host system. Unlike traditional virtual machines, containers share the host operating system kernel, resulting in minimal performance overhead while maintaining environment consistency. Container platforms such as Docker have significantly simplified software deployment across many domains of scientific computing (Pahl, 2015). However, Docker typically requires administrative privileges, limiting its applicability on shared HPC clusters.

To address this limitation, Singularity was developed specifically for HPC environments (Kurtzer et al., 2017). Singularity enables users to execute containerized applications without requiring root privileges, making it well suited for multi-user computing clusters. It also integrates naturally with HPC technologies such as distributed file systems, message passing interfaces (MPI), and job scheduling systems, thereby facilitating the deployment of complex scientific workflows across large-scale computing infrastructures.

Despite the growing adoption of container technologies in scientific computing, their use in computational neuroscience, particularly for large-scale spiking neural network simulations, remains relatively limited. Concerns have been raised regarding potential performance overhead, compatibility with MPI-based distributed simulations, and efficient utilization of GPU hardware. Previous studies have demonstrated that GPU acceleration can substantially improve the performance of neural simulations (Deistler et al., 2025; Knight and Nowotny, 2021; Kumbhar et al., 2019; Yavuz et al., 2016; Zenke and Gerstner, 2014; Zhang et al., 2023) yet integrating such capabilities within portable and reproducible computational environments remains an ongoing challenge. Furthermore, many established neural simulation frameworks were originally developed prior to the widespread adoption of container technologies and remain tightly coupled to specific software configurations.

In this study, we present a container-based computational framework designed to support scalable and reproducible neural simulations on modern HPC systems. The proposed framework encapsulates the complete simulation environment, including compilers, numerical libraries, MPI runtimes, GPU toolchains, and neural simulation software, within a portable container image. By leveraging Singularity containers together with distributed MPI execution and GPU acceleration, the framework enables large-scale spiking neural network simulations to be deployed consistently across heterogeneous HPC infrastructures.

To evaluate the effectiveness of this approach, we conducted benchmarking experiments using a network model, created by replicating a cerebellar granular layer module comprising mossy fiber and granule cell populations. We chose this model since the cerebellum is an ideal target for the following reasons: It contains the most abundant type of neuron in the whole mammalian brain, the cerebellar granule cell (GrC). Due to their high density, the size of a granular layer circuit model grows quickly with its anatomical scale. A large scale is also necessary because the postsynaptic targets of GrCs, the cerebellar Purkinje cells, each receive input from ∼100,000 GrCs. Notably, biologically well-constrained cell models (Solinas et al., 2010; Sudhakar et al., 2017) and connectome data (Nguyen et al., 2023) are both available. While the publicly available connectome data can translate to a large network model (Nguyen et al., 2023), we created an even larger-scale simulation workload by replicating this base model multiple times, resulting in systems containing millions of neurons and synaptic connections.

Using this model, we assessed the computational overhead, scalability, and cross-system reproducibility of our container-based approach. We evaluated the framework on two systems that differ in both CPU and GPU generation. On the benchmark system, we measured the cost of containerization directly, by running an otherwise identical native toolchain on the same node, and we characterized GPU scaling from one to three devices. On the second system, we verified that the same container image runs unmodified and reproduces the simulation output exactly.

Our results demonstrate that, compared to native installations, containerized simulations can suffer performance overhead only up to ∼1% while providing exactly reproducible results on top of significant advantages in portability. These findings suggest that container-based workflows offer a practical foundation for large-scale computational neuroscience, enabling researchers to deploy complex simulation environments across diverse computing infrastructures with minimal configuration effort and easily share their work for reproducibility.

## 2 Materials and methods

### 2.1 Overview of the container-based framework

**FIGURE 1:**
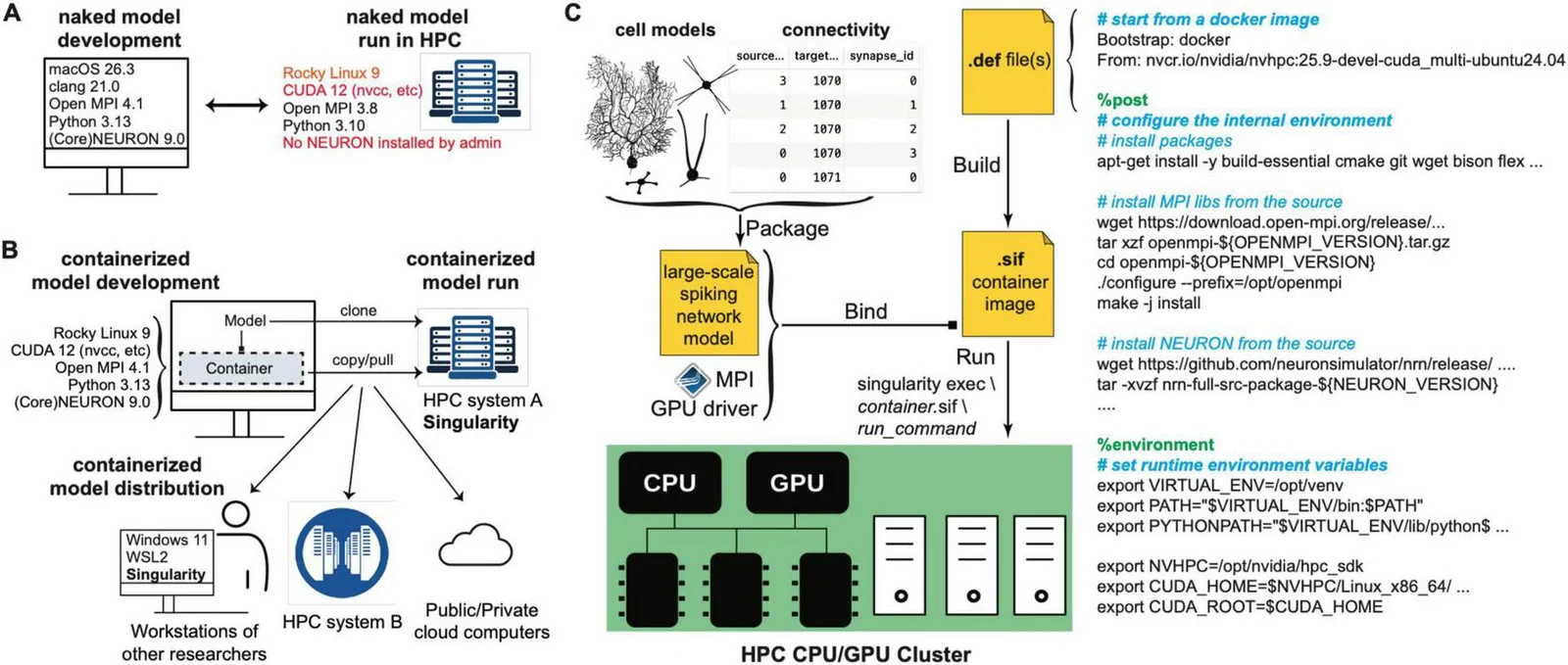
Singularity container-based framework for portable large-scale neural network simulations on HPC infrastructure. **(A)** Traditional “naked” model development. Researchers develop their models in fixed environments in their workstations and/or HPC servers while their simulation environment can be partially (orange) or completely incompatible (red). **(B)** Containerized development. The container packages all required software dependencies including compilers, simulation platforms, GPU drivers, and other libraries into a portable execution environment without requiring root access on the host system. The model codes can be cloned from repositories or also packaged into the containers. Distributing the model with the container reproduces the simulation results on the systems that we tested. **(C)** Our framework. A definition (.def) file specifies the full software stack (right). Then, a container image (.sif) encapsulating the reusable, entire computational environment is built. Large-scale neural network models, packaged into model definition files in NeuroML2 (Cannon et al., 2014), SONATA (Dai et al., 2020), etc., are bound to the container image at runtime. The container can also bind to the MPI-based parallel execution and GPU acceleration of the host system. Then, the containerized model can run consistently regardless of the underlying hardware configuration, ensuring reproducibility and portability.

Our central idea is that applications for developing large-scale spiking network models (simulators, scaffold builder, etc.) and their complex software dependencies are encapsulated within a portable execution environment, the so-called container image. Then, the models in development and container images are distributed together to HPC systems other than those that researchers originally used (Figure 1). This approach allows the same simulation environment to be deployed consistently across different HPC systems without requiring modifications to the host software stack.

Specifically, we built the framework using Singularity, the container platform designed for HPC systems. In particular, we used the open-source version of Singularity that is also called Apptainer^1^. Since our focus is developing the biologically grounded large-scale spiking neural network, the framework integrated the CoreNEURON (Kumbhar et al., 2019) and its dependencies for distributed and GPU-based computing for simulating large-scale spiking neural network models. Therefore, the container image was defined and built with all required software components, including compilers, numerical libraries, MPI runtime environments, GPU-enabled backends, and CoreNEURON, by describing the specifications and installation steps in Singularity definition file (.def file; Sylabs Inc, 2024a; Sylabs Inc., 2024b; Figure 1C). Using the Singularity build command (Sylabs Inc, 2024a) the definition file is compiled into a portable.sif image file. Once built and validated, the .sif container image can be copied and reused across different computational infrastructures while maintaining consistent execution behavior (Figure 1B).

Model development typically involves many cycles of iterations for improvements and bug fixes, which often requires reconfiguration of the software dependencies. Notably, the container images are static and any changes in the software stack in them require rebuilding the images with modified .def files, which seems to impose difficulty in the continuous model development process. However, this issue can be managed in two ways.

The first approach is building the container images in multiple steps. The Singularity definition files can be defined based on other images and therefore one can define the full stack via multiple .def files. For example, our framework is built through three different .def files: the first one starts from the NVIDIA HPC SDK 25.3 with Ubuntu 24.04 base docker image and installs the distributed computing packages UCX and OpenMPI in the image. The second one installs the uv Python package managing system for managing scientific Python packages that CoreNEURON depends on. The last one installs CoreNEURON on top of the last two steps.

The second approach exploits Singularity’s ability to bind the files/directories between the images and the host. In this way, one can still continuously update the model codes while running simulations as if the model is residing in the container image. Many simulation platforms, such as CoreNEURON, require compilation of the mechanism definitions (e.g., MOD files) before running simulations, but this step also can be handled by compiling the mechanisms by the compilers within a container image and binding the compiled binary outputs (e.g., libcorenrnmech.so).

Table 1 shows the hardware and software specification used in this study. For more details, please see our code repository https://github.com/NeuroAI-IBS/singularity_spiking_network.

**TABLE 1:** Hardware and software specification of the two systems.

Component	Benchmark system (“Eren”)	Portability system (“Zeke”)
CPU	2 × Intel Xeon Silver 4210R (Cascade Lake, AVX-512) @ 2.40 GHz (max 3.20 GHz); 20 physical cores/40 threads; 2 NUMA nodes	Intel Core i7-6850K (Broadwell-E, no AVX-512) @ 3.60 GHz; 6 physical cores/12 threads; 1 NUMA node
GPU	3 × Tesla V100-SXM2-32GB (compute capability 7.0); driver 580.173.02 (CUDA driver API 13.0)	Quadro GP100 (6.0, not used) + 3 × TITAN Xp (6.1, used); driver 580.173.02 (CUDA driver API 13.0)
Interconnect	Single node; shared-memory MPI (no InfiniBand fabric)	Single node; shared-memory MPI
Toolchain	NVIDIA HPC SDK 25.3 (CUDA 12.8); UCX 1.17.0; Open MPI 4.1.8 (internal PMIx, UCX transport); Python 3.12.3; uv 0.11.21; NEURON 9.0.1 with CoreNEURON (GPU enabled, OpenACC via NVHPC)	Identical, supplied by the same container image copied from the benchmark system
Native arm	Environment Modules 5.4.0	Not applicable (container arm only)
Container runtime	Apptainer 1.5.3	Apptainer 1.5.3
Runtime settings	NVIDIA CUDA Multi-Process Service (MPS) enabled; no explicit MPI rank binding	Same as benchmark system
Scheduler	None; runs launched directly on a dedicated node	None; runs launched directly on a dedicated node

All timings reported in this study were measured on Eren. On Zeke the entire software stack is supplied by the container image copied from Eren; nothing was installed on the host.

### 2.2 Cerebellar network model

We employed a biologically grounded cerebellar circuit model comprising mossy fibers (MFs) and granule cells (GrCs). The baseline network architecture was derived from published anatomical data (Nguyen et al., 2023) and the cell models were from our previous study (Sudhakar et al., 2017).

Briefly, the modeled circuit represents the feedforward input stage of the cerebellar cortical network, where MFs provide excitatory input to GrCs through glutamatergic synapses. GrCs receive convergent inputs from multiple MFs and generate spiking responses. In our simulations, neurons were modeled using conductance-based spiking models taken from a previous study (Sudhakar et al., 2017) and adapted to the CoreNEURON platform for optimized large-scale performance. The baseline network consists of 1,070 mossy fibers and 3,925 granule cells with 12,387 synaptic connections (Nguyen et al., 2023). The model GrCs incorporated a comprehensive set of ion channel and synaptic conductance mechanisms including voltage-gated sodium (Na, NaR, PNA), potassium (KV, KA, KM, KIR, KCA), and calcium (CA, CALC) channels, alongside tonic GABAergic inhibition and NMDA leak conductances (Sudhakar et al., 2017).

To generate computational workloads suitable for large-scale benchmarking, we constructed larger networks by replicating this base circuit 280 times. Specifically, independent copies of the MF-GrC network were instantiated within the same simulation domain. Each replicated module maintained identical connectivity structure and neuronal dynamics but operated independently without cross-module synaptic interactions. This approach preserves the biological characteristics of the original circuit while enabling controlled scaling of network size and computational complexity. The final network model contained 299,600 MFs, 1,099,000 GrCs, and 3,468,360 synapses.

We ran 300 ms simulations. Random spike trains from MFs mimicking background baseline activity (10 Hz) were used to drive GrC excitatory inputs. Because the replicated networks are independent, this configuration provides an effective method for stressing computational resources while preserving realistic neuronal dynamics. The resulting large-scale simulations therefore serve as a practical benchmark for evaluating distributed parallel execution and GPU acceleration within the containerized HPC framework. More details about the simulation parameters are listed in Supplementary Table 1.

We conducted our experiments in seven different arms (configurations) on the benchmark system (“Eren,” see Table 1). Those arms are designed to measure, first, the container overhead compared to native CPU only/GPU runs and, second, GPU acceleration scaling in container runs. We also ran three different configurations on the portability test system (“Zeke,” see Table 1) for testing the portability of the GPU runs via the same container image files copied from Eren. The random seed was held fixed and the configurations were interleaved run by run (Supplementary Table 1). We evaluated the difference as a paired per-repetition difference, with *n* = 5 after discarding the first repetition as a warm-up. A comprehensive list of all the arms, parameters, and rationale can be found in Supplementary Table 2.

## 3 Results

We constructed a Singularity container image with a focus on running a large-scale spiking network model on the CoreNEURON simulation engine (Kumbhar et al., 2019) with MPI-based distributed computing and GPU-based acceleration and also developed a large-scale spiking network grounded on the cerebellar granular layer network comprising ∼1 million neurons (see Methods). Then, we performed three computational benchmark experiments to address the questions regarding efficiency, scalability, and portability of the container-based framework as follows:

- Experiment 1, cost of containerization: The native and containerized simulations on our benchmark system, “Eren” (see Table 1), are compared at matched rank and device counts: CPU at 18 MPI ranks, and GPU at 18 ranks with three devices. The only variable is containerization.
- Experiment 2, scaling of GPU acceleration: The containerized CPU configuration at 20 MPI ranks, which is the maximum allowed in Eren, is compared with the containerized GPU configurations at one, two and three devices. The only variable is the number of the compute devices, and all the runs were made via the container.
- Experiment 3, running the same simulation in a different system: The container image file is copied to a portability test system, “Zeke” (see Table 1) to run the same model. The only variable is the HPC system.

**FIGURE 2:**
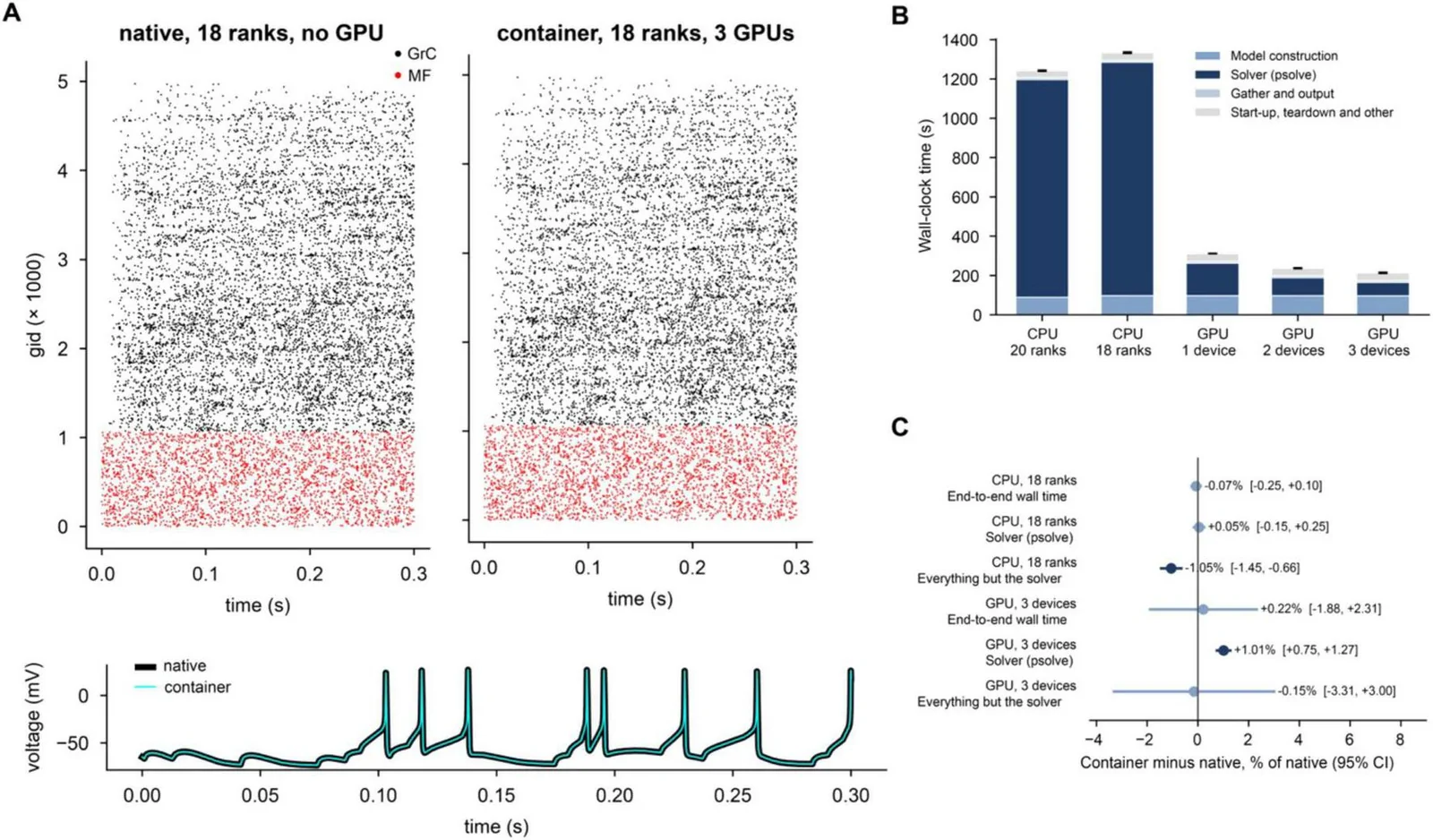
Containerization changes neither the result nor the run time. **(A)** Top, spike raster of one base module for a native 18-rank CPU run (left) and a containerized 18-rank three-GPU run (right). Red: MFs (gid = 0–1069). Black: GrCs (gid = 1070–4994). Bottom, membrane potential of one GrC (gid = 1080) from the same two runs, superimposed. **(B)** Composition of the total wall time for the five containerized configurations. Bars are means over *n* = 5 repetitions; the error bar is SD. **(C)** Container minus native, paired per repetition, as a percentage of the native mean with 95% CI (*n* = 5). Positive means the container is slower. Dark points mark intervals excluding zero.

Every experiment simulated the same 300 ms of activity in the replicated cerebellar granular layer network (Figure 2A). In the following sections, we explain the results of those three experiments one by one.

### 3.1 Experiment 1–cost of containerization is minimal

The first experiment asks the cost of container-based simulations compared to native ones. To answer it, we built a native, non-containerized toolchain on the benchmark system that matches the container image layer for layer as described in Table 1. We additionally confirmed that the two arms carry identical compiler provenance, in that the compiler version strings embedded in the compiled mechanism library are the same in both. Both arms then ran on the same node, with the same model, the same number of MPI ranks, the same GPUs and the same Multi-Process Service (MPS) configuration.

We found that containerized and native execution were indistinguishable in total wall time. The paired difference was −0.98 s (−0.07%, 95% CI [−0.25%, +0.10%]) in the CPU configuration and +0.46 s (+0.22%, 95% CI [−1.88%, +2.31%]) in the three-GPU configuration (Figure 2C and Table 2). The overhead of a container can arise in image mounting, dynamic library resolution, MPI initialization and bind-mounted filesystem access. The distinction between solver time and total wall time matters here because the two configurations we compared devote very different fractions of their run to the solver: 1187.98 s of a 1333.70 s run in the CPU configuration, but only 68.32 s of 213.90 s in the three-GPU configuration (Supplementary Tables 3, 4).

**TABLE 2:** Experiment 1, cost of containerization.

Configuration	Metric	Native (s)	Container (s)	Difference	Resolved
CPU, 18 ranks	Total wall time	1334.68 ± 1.07	1333.70 ± 2.76	−0.98 s (−0.07%) 95% CI [−0.25%, +0.10%]	No
CPU, 18 ranks	Solver (psolve)	1187.41 ± 1.22	1187.98 ± 2.64	+0.57 s (+0.05%) 95% CI [−0.15%, +0.25%]	No
CPU, 18 ranks	Everything except solver	147.28 ± 0.30	145.73 ± 0.34	−1.55 s (−1.05%) 95% CI [−1.45%, −0.66%]	Yes
CPU, 18 ranks	Start-up interval	27.19 ± 0.07	28.02 ± 0.09	+0.82 s (+3.03%) 95% CI [+2.78%, +3.28%]	Yes
GPU, 18 ranks, 3 GPUs	Total wall time	213.44 ± 2.44	213.90 ± 2.01	+0.46 s (+0.22%) 95% CI [−1.88%, +2.31%]	No
GPU, 18 ranks, 3 GPUs	Solver (psolve)	67.64 ± 0.13	68.32 ± 0.16	+0.69 s (+1.01%) 95% CI [+0.75%, +1.27%]	Yes
GPU, 18 ranks, 3 GPUs	Everything except solver	145.80 ± 2.41	145.58 ± 2.11	−0.22 s (−0.15%) 95% CI [−3.31%, +3.00%]	No
GPU, 18 ranks, 3 GPUs	Start-up interval	27.33 ± 0.19	28.65 ± 0.50	+1.31 s (+4.81%) 95% CI [+2.36%, +7.25%]	Yes

Paired per-repetition difference on the benchmark system, container minus native, at matched rank and device counts (*n* = 5). Percentages are relative to the native mean. Positive: the container is slower. Resolved: the 95% CI excludes zero.

Two smaller differences are nevertheless resolved: First, the containerized solver on three GPUs is 1.01% slower than the native one (+0.69 s, 95% CI [+0.75%, +1.27%]). This is detectable only because the GPU solver is highly reproducible, with a standard deviation of 0.13–0.16 s over five repetitions while the same effect is invisible in total wall time, whose standard deviation is more than an order of magnitude larger. Second, everything except the solver is 1.05% faster inside the container in the CPU configuration (−1.55 s, 95% CI [−1.45%, −0.66%]). The two effects run in opposite directions and neither is apparent in the end-to-end number.

We localized the remaining container cost by separately timing the interval that precedes the simulator’s own logging, which contains process launch, interpreter start-up on all ranks, GPU context creation and opening of the model database, which is precisely where the cost of starting a container would appear. This interval is the one place where our measurements do resolve such a cost: 27.19 ± 0.07 s natively versus 28.02 ± 0.09 s in the container in the CPU configuration (+0.82 s, +3.03%, 95% CI [+2.78%, +3.28%]), and 27.33 ± 0.19 s versus 28.65 ± 0.50 s on three GPUs (+1.31 s, +4.81%, 95% CI [+2.36%, +7.25%]). In absolute terms this amounts to roughly one second, which is 0.06% of the CPU run and 0.61% of the three-GPU run. Notably, it is a fixed cost paid once per invocation and does not grow with the duration of the simulation, so its relative weight falls as simulations become longer.

Finally, containerization changed neither the result nor, at the resolution of our measurements, the time required to obtain it. The native and containerized arms produced bit-identical spike output; these comparisons are among the 15 pairwise tests reported in Supplementary Table 5, all of which matched exactly.

### 3.2 Experiment 2–GPU acceleration boosts performance, scaling with GPUs

In this experiment, we took the containerized CPU configuration at 20 MPI ranks, the maximum number of physical cores available in Eren, as the baseline. We verified by measurement that this is also the faster of the two CPU configurations we ran: at 20 ranks the solver is 6.75% faster than at 18 ranks (−80.13 s, 95% CI [−81.74, −78.52] s) and the total wall time is 6.88% faster (Supplementary Table 6). Adopting the faster CPU configuration means that the speed-up reported below is a conservative one. All the runs compared here were made through the container, so that the only variable is the number of compute devices.

**FIGURE 3:**
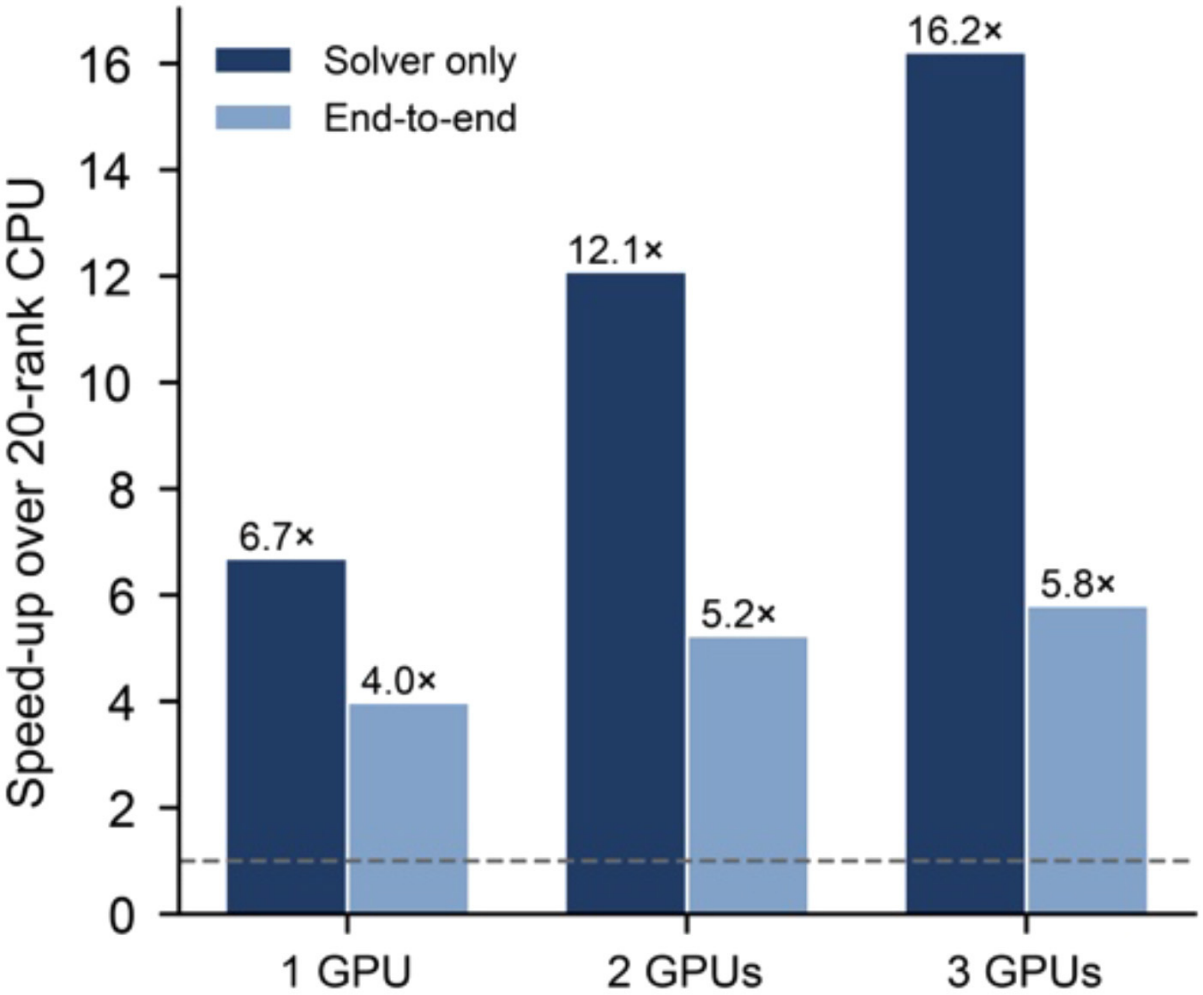
Speed-up scales with GPU count. Speed-up over the 20-rank CPU baseline for one, two and three GPUs. Dark: solver (psolve) time. Light: end-to-end wall time. Dashed line: baseline. All runs are containerized. Means over *n* = 5 repetitions.

Adding GPUs reduced the solver time sharply and monotonically. The solver required 1107.85 s on 20 CPU ranks, 165.53 s on one GPU, 91.65 s on two, and 68.32 s on three (Supplementary Table 3). Relative to the CPU baseline, three GPUs therefore accelerate the solver by 16.21× (Figure 3 and Supplementary Table 7). Scaling across devices is sublinear, as is expected at a fixed problem size: two GPUs are 1.81× faster than one, and three are 2.42× faster than one.

On the other hand, the total wall time falls from 1241.95 s to 213.90 s, which is a speed-up of 5.81× rather than 16.21×, and the improvement from one to three GPUs is 1.46×. The reason is that only the solver is accelerated (Figure 2B). In the three-GPU configuration the solver accounts for 68.32 s of a 213.90 s run, while the remaining 145.58 s (68.1%) is spent on model construction (96.35 s), process start-up and teardown (39.69 s), and output gathering and writing (9.53 s). This unaccelerated remainder is essentially constant across the GPU configurations, at 146.20 s, 145.42 s and 145.58 s for one, two and three devices respectively, and it therefore imposes a floor on the end-to-end time that additional devices cannot lower. Model construction, which dominates that floor, is also the only stage with appreciable imbalance between ranks: cell creation varies by up to 5.2% and synapse creation by up to 2.8% across the 18 ranks (Supplementary Table 8).

In summary, the container framework still delivered a performance boost, which scaled with the number of GPUs used. With the maximal number of GPUs used, the container-based simulations ended up suffering from a tiny overhead of ∼1% in the solver part while this difference can be overwhelmed by other factors determining the total wall time.

### 3.3 Experiment 3–container image delivers reproducible results across different systems

In the third part, we tested portability by simply copying the container image file from Eren to Zeke, a machine very different from Eren in both CPU and GPU generation (Table 1). Notably, no toolchain software on Table 1 was installed on the host, and the image was neither rebuilt nor modified. Then, we ran the GPU arm there with 6 MPI ranks, matching the six physical cores of Zeke.

**FIGURE 4:**
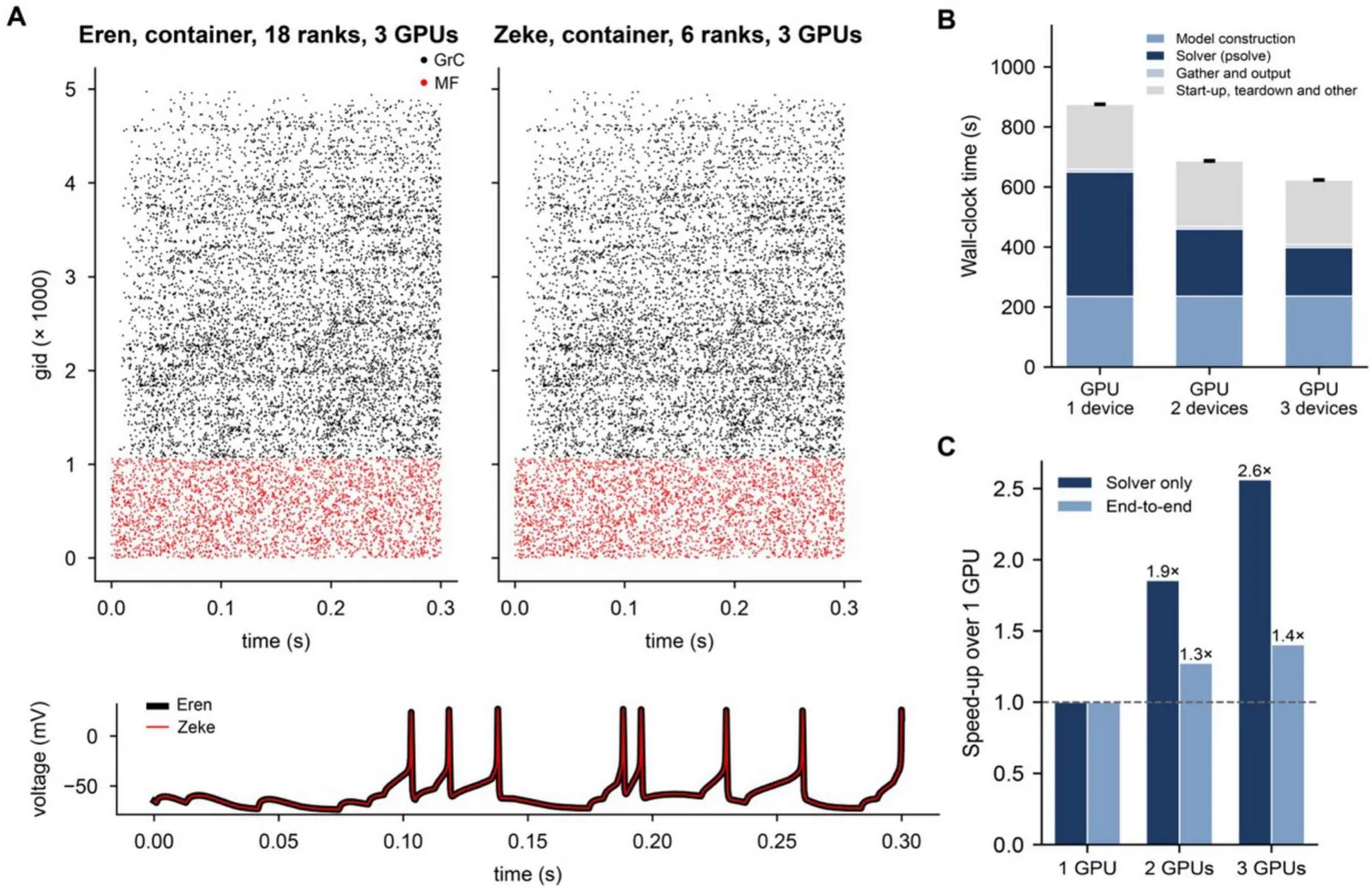
The same container image reproduces the computation on a second machine. **(A)** Same spike raster (Top) and membrane potential (Bottom) comparison as Figure 2A between a containerized 18-rank three-GPU run on Eren and a containerized 6-rank three-GPU run on Zeke. **(B)** Same as Figure 2B for Zeke runs. Bars are means over *n* = 5 repetitions; the error bar is SD. **(C)** Same as Figure 3 for Zeke runs. Note that the baseline is now the 1 GPU case.

While the image ran unmodified, the simulation produced the same results as in Eren (Figures 4A–C): the simulations were accelerated by GPUs successfully with performance scaling with the number of GPUs (Supplementary Tables 9, 10). The spike outputs were identical (Supplementary Table 11). This result demonstrated the strength of our container-based framework in the ease of portability while maintaining reproducibility.

We note that this exactness is structural rather than accidental. In CoreNEURON, each cell is integrated independently within a spike-exchange interval (Hines et al., 2008). Therefore, there is no floating-point reduction whose summation order changes when the work is redistributed over a different number of ranks or devices, and the exchange interval itself is held fixed. With the random seed held constant, the computation is deterministic in the strict sense that the same bits are produced on different hardware, and not merely statistically equivalent.

We also discovered the limitations of portability: our first image, built on Eren, the Cascade Lake host, aborted with an illegal-instruction fault on Zeke, which lacks AVX-512. This demonstrates that containerization fixes only the software stack and cannot make computers run binary codes that are incompatible with their hardware. The same consideration applies to GPU code, which must be compiled for every compute capability on which the image is expected to run. We will discuss this point in the Discussion.

Despite this issue, our experiment showed that the container-based framework can make distributing and deploying large-scale spiking neural network models across systems with diverse configurations as easy as copying a single file from one system to another.

### 3.4 Conclusion

The three experiments are independent by design, but they combine into a single statement. Experiment 2 measures GPU acceleration entirely within the container, and Experiment 1 shows that at matched rank and device counts the container is indistinguishable from a native toolchain on the same node; the acceleration is therefore a property of the toolchain and the hardware, not of the container, and the two results can be read together. Experiment 3 shows that the same image, carried to a machine of another CPU and GPU generation, computes the same result. Taken together, the framework can be adopted without paying for it in run time and without changing what is computed.

## 4 Discussion

The increasing scale and complexity of computational neuroscience models have made high-performance computing (HPC) essential for realistic simulations of biological neural networks, and at the same time, the heterogeneity of modern HPC infrastructures, such as differences in operating systems, compiler toolchains, runtime libraries, and GPU environments has made complex simulation frameworks difficult to deploy across different institutions (Pahl, 2015). In this study, we addressed these challenges by developing a container-based framework for large-scale neural simulations and evaluating it on a biologically grounded cerebellar base module replicated for benchmarking. Using containerization based on Singularity, we encapsulated the entire simulation stack, including compilers, MPI libraries, GPU toolchains, and the simulation platform, within a portable execution environment. In this way, the same simulation configuration can be executed on another system without modifying its host software stack. Measured against an otherwise identical native toolchain on the same node, this cost no difference in total wall time that our measurements could resolve; three GPUs shortened the solver by 16.21× and the complete run by 5.81×; and the same image, copied to a machine of another CPU and GPU generation, reproduced the output exactly. Containerized workflows are therefore a practical foundation for large-scale computational neuroscience, not because they are fast, but because what they cost is now measured rather than assumed.

We performed three experiments built around a single variable, and that is what allows the contribution to be stated narrowly. The first one bounds the cost of containerization on one machine, against a native toolchain matched layer for layer; it does not, however, establish native-equivalent performance in general. The second one characterizes GPU scaling within the container on that machine, over one to three devices. The third experiment shows that an image carried to a machine of another hardware generation computes the same thing. Stating the contribution at this width is what the design of the experiments supports; anything broader would require machines, interconnects and models we did not test.

Our analysis of the computational cost of containerization shows that it does not slow the integration down step by step. Instead, it charges once, at start-up, for mounting the image, resolving dynamic libraries and initializing MPI. This fixed invocation cost divides out over a long run but dominates a short one. Therefore, workflows built from many short invocations, such as parameter sweeps, unit tests, interactive debugging, and benchmarking like this study, will suffer from it while a single production run of hours will not. This also explains why the one difference we could resolve inside the solver, of about one percent on GPUs, is of little practical consequence: it is smaller than the run-to-run variation of the total wall time, and it moves in the opposite direction to a comparable effect outside the solver. In our case, we used a simulated duration of 300 ms to keep the benchmark matrix tractable, whereas the questions this model is built for would usually be posed over much longer biological time. Since solver time scales with that duration while construction and start-up do not, our benchmark understates the end-to-end factor relative to what a longer simulation would give. Solver time is the quantity usually quoted in this literature (Knight and Nowotny, 2021; Kumbhar et al., 2019; Zhang et al., 2023), and it remains the right one for comparing integration schemes. Our breakdown adds that the two coincide only when the solver dominates: for short runs, and for workflows that invoke the simulator often, the unaccelerated remainder, particularly model construction, is where the next optimization lies.

We particularly note two limitations that bound the timing results. All measurements were made on single, dedicated nodes with shared-memory MPI. We do not extend these conclusions to multi-node execution, to other interconnects, or to other schedulers, none of which were tested here, and container-MPI interaction across a fabric such as InfiniBand is precisely the setting in which overhead is most likely to appear, since it is the one place where the container must negotiate with hardware the host controls. Separately, the container–native equivalence was established at 18 MPI ranks, whereas the CPU baseline for the GPU comparison was measured at 20. We expect the equivalence to hold there since the container cost arises in filesystem access and process launch and is independent of rank count, but this is an assumption rather than a measurement.

Our benchmark network model, created by replicating an anatomically constrained biologically grounded module of the cerebellar granular layer network, was designed to produce a workload of the right size and numerical character to stress the hardware. Therefore, it should be considered a computational stress test rather than a simulation with physiological realism. With this design, we could avoid modeling decisions that would confound the timing comparison, but it limits what the timings predict. A circuit of similar size with Golgi cells, Purkinje cells and molecular layer interneurons would carry recurrent and inhibitory pathways, and therefore a different balance between local computation and spike exchange. Then, the shape of the GPU scaling curve would inevitably change. Extending the framework in that direction is the natural next step.

From the portability perspective, it is notable how little must exist on the target machine in the container-based framework. Our own runs were launched directly on dedicated nodes, and we did not test this in a scheduled cluster environment, but the requirement is in principle modest. Since the MPI stack lives inside the image, a launcher that uses a process-management interface such as PMI2 or PMIx, just like Slurm’s srun, can wire up the containerized ranks without a matching MPI installation on the host. Similarly for GPU execution, the host needs its NVIDIA kernel driver and the matching user-space driver libraries, which the container runtime locates and binds in. However, neither the CUDA toolkit nor the compiler suite used to build the image, such as the NVIDIA HPC SDK, has to be installed there. What a site must supply therefore reduces to the container runtime, a PMI-capable launcher, and a sufficiently new GPU driver for the CUDA version that the image was built against.

However, one lesson from the portability experiment generalizes beyond this study. Containerization fixes the software stack, but it does not by itself make a simulation portable across hardware generations. Compilers target the machine they are invoked on unless told otherwise, and therefore an image built with default flags embeds the instruction set of the build host. The same applies to GPU code, which must be compiled for every compute capability on which the image is expected to run. Building for a common instruction-set baseline and for the full range of compute capabilities in use is therefore not an optimization detail but a precondition for the image to run at all elsewhere. We note this because the problem is invisible until the image reaches a second machine, and because it is the difference between an image that is merely self-contained and one that is genuinely portable. For the same reason, we emphasize that what our third portability experiment demonstrates is evidence that the image reproduces the computation, not that it reproduces the run time.

Potentially the most important feature of the container-based framework is that it makes it easy to version the execution environment and the science separately, since the model layer is bound at run time rather than built into the image. A typical dilemma in conducting reproducible modeling research is that reproducibility favors freezing everything whereas model development requires changing something almost daily. Here the environment can be frozen and archived in the container image while connectivity, mechanisms, and parameters can continue to change, which also applies to pathology-driven modifications to the model. The same separation is what makes an archived result re-executable years later, when the original software would no longer build on a current system (Stodden et al., 2016): the image or its definition files (Boettiger, 2015) supply the environment, and only the model files distinguish one study from another.

This also determines how the framework relates to the tools that build circuits in the first place. Packages such as the Brain Scaffold Builder (BSB; De Schepper et al., 2022) and Pycabnn (Wichert et al., 2020) address how a biologically realistic circuit is specified and generated. In particular, BSB has since been extended beyond its original target system, the cerebellum, and coupled to mean-field and whole-brain approaches (Lorenzi et al., 2025; Sheiban et al., 2026). What we address by the container-based approach is the step after that, executing a constructed circuit reproducibly on hardware the modeler does not control. Therefore, the two are complementary rather than competing. BSB or similar packages can leverage the container-based approach in two ways, depending on how settled the builder is. A builder still under development can be bound to the image at run time, so that it changes without the image having to be rebuilt. A builder that has stabilized can instead be installed into the image and distributed with it, entirely removing the installation step for downstream users.

In summary, this study offers a practical and efficient solution for deploying large-scale spiking neural network models on modern HPC systems. The proposed framework provides fixed and versioned simulation execution environments, which are both portable and computationally efficient, according to our measurements. As computational neuroscience models continue to increase in scale and complexity, such approaches will be essential for ensuring that simulation workflows remain portable, reproducible, and accessible to the broader research community.

## Data Availability

The datasets presented in this study can be found in online repositories. The names of the repository/repositories and accession number(s) can be found below: https://github.com/NeuroAI-IBS/singularity_spiking_network and doi: 10.5281/zenodo.21828283.

## Footnotes

1
https://apptainer.org
