# Supplementary material for: Container-based framework for large-scale spiking network simulation
Source: Front Neurosci. 2026 Sep 9;20:1893064. doi: 10.3389/fnins.2026.1893064 (PMC13597816; doi:10.3389/fnins.2026.1893064)
Supplement: Supplementary file 1 [file Supplementary_file_1.docx]

Supplementary Material

# All timings in this document were generated from the deposited per-run logs by the deposited analysis script; no value is transcribed by hand. Unless stated otherwise, each configuration was run six times, the first run discarded as a warm-up, and the remaining five analyzed (n = 5). Per-phase values are those reported by the master MPI rank (rank 0), then means and standard deviations over repetitions. Total wall time is measured by the launching script around the entire invocation and therefore includes process start-up, model construction, solver, output gathering and file writing.

# 1 Supplementary Figures and Tables

## 1.1 Supplementary Tables

| **Parameter** | **Value** | **Note** |
| --- | --- | --- |
| Simulated duration (tstop) | 300 ms | Identical in all configurations |
| Integration step (dt) | 0.025 ms | Fixed step |
| Spike-exchange interval (maxstep) | 10 ms | CoreNEURON communication interval |
| Random seed (global_seed) | 0 | Held fixed; repetitions measure machine variability, not model variability |
| Repetitions per configuration | 6 recorded, 5 analyzed | First repetition discarded as warm-up; retained in the deposited raw data |
| Run ordering | Interleaved | Configurations interleaved rather than blocked, so thermal drift is spread equally across arms |
| MPI rank binding | None | Measured to make no difference |
| Wall-time scope | Launching script | Includes start-up, construction, solver, gathering and output |

**Supplementary Table S1.** Simulation and timing protocol. Every parameter listed was held fixed across all repetitions, all configurations and both systems, so that the only quantities differing between runs are the ones under test: execution arm, MPI rank count and GPU count.

| **Configuration** | **System** | **Arm** | **Ranks** | **GPUs** | **Runs** | **Why this configuration was run** |
| --- | --- | --- | --- | --- | --- | --- |
| container_cpu_r20_g0 | Eren | Container | 20 | — | 6 (5) | CPU baseline for Experiment 2. Uses every physical core of the machine and is the faster of the two CPU rank counts, so it is the conservative denominator for the GPU speed-up. |
| container_cpu_r18_g0 | Eren | Container | 18 | — | 6 (5) | Container side of the Experiment 1 CPU pair, rank-matched to native_cpu_r18_g0. Also reported as the alternative CPU baseline. |
| container_gpu_r18_g1 | Eren | Container | 18 | 1 | 6 (5) | GPU scaling point, one device (Experiment 2). |
| container_gpu_r18_g2 | Eren | Container | 18 | 2 | 6 (5) | GPU scaling point, two devices (Experiment 2). |
| container_gpu_r18_g3 | Eren | Container | 18 | 3 | 6 (5) | GPU scaling end point (Experiment 2) and container side of the Experiment 1 GPU pair. |
| native_cpu_r18_g0 | Eren | Native | 18 | — | 6 (5) | Native side of the Experiment 1 CPU pair. Same node, same model, same rank count as the container CPU arm. |
| native_gpu_r18_g3 | Eren | Native | 18 | 3 | 6 (5) | Native side of the Experiment 1 GPU pair. Same node, same model, same rank and device count as the container GPU arm. |
| container_gpu_r6_g1 | Zeke | Container | 6 | 1 | 6 (5) | Portability check on the second machine, one device (Experiment 3). Six ranks matches that machine’s six physical cores. |
| container_gpu_r6_g2 | Zeke | Container | 6 | 2 | 6 (5) | Portability check on the second machine, two devices (Experiment 3). |
| container_gpu_r6_g3 | Zeke | Container | 6 | 3 | 6 (5) | Portability check on the second machine, three devices (Experiment 3). |

**Supplementary Table S2.** Every configuration run in this study, and why it was run. The Runs column gives the number of recorded repetitions and, in parentheses, the number analyzed — the first repetition of each configuration is a warm-up and is excluded.

| **Phase (s)** | **Container CPU, r20** | **Container CPU, r18** | **Native CPU, r18** | **Container GPU, 1** | **Container GPU, 2** | **Container GPU, 3** | **Native GPU, 3** |
| --- | --- | --- | --- | --- | --- | --- | --- |
| Cell creation | 24.024 ± 0.199 | 26.002 ± 0.167 | 26.152 ± 0.331 | 26.006 ± 0.339 | 25.973 ± 0.105 | 25.852 ± 0.225 | 26.146 ± 0.138 |
| Synapse creation | 59.079 ± 0.403 | 64.618 ± 0.628 | 65.658 ± 0.598 | 64.740 ± 0.368 | 64.650 ± 0.610 | 64.093 ± 0.514 | 65.036 ± 0.319 |
| Connection creation | 2.778 ± 0.008 | 3.013 ± 0.020 | 3.003 ± 0.029 | 3.014 ± 0.022 | 3.028 ± 0.035 | 3.001 ± 0.031 | 3.017 ± 0.019 |
| Cell parameter application | 0.496 ± 0.008 | 0.489 ± 0.003 | 0.498 ± 0.002 | 0.490 ± 0.002 | 0.487 ± 0.005 | 0.486 ± 0.003 | 0.491 ± 0.007 |
| Spike recording setup | 2.562 ± 0.019 | 2.836 ± 0.003 | 2.884 ± 0.034 | 2.840 ± 0.016 | 2.848 ± 0.014 | 2.835 ± 0.013 | 2.841 ± 0.041 |
| Voltage recording setup | 0.084 ± 0.001 | 0.087 ± 0.000 | 0.091 ± 0.000 | 0.087 ± 0.001 | 0.087 ± 0.001 | 0.087 ± 0.001 | 0.091 ± 0.000 |
| **Simulation (psolve)** | **1107.847 ± 2.474** | **1187.977 ± 2.645** | **1187.409 ± 1.218** | **165.527 ± 0.128** | **91.646 ± 0.071** | **68.322 ± 0.158** | **67.637 ± 0.130** |
| Data gathering (alltoall) | 3.452 ± 0.032 | 3.479 ± 0.018 | 3.476 ± 0.024 | 3.478 ± 0.022 | 3.484 ± 0.055 | 3.458 ± 0.064 | 3.442 ± 0.039 |
| Data merging | 6.005 ± 0.108 | 5.991 ± 0.041 | 6.091 ± 0.090 | 5.989 ± 0.031 | 6.044 ± 0.032 | 6.002 ± 0.059 | 6.046 ± 0.098 |
| Output save | 0.071 ± 0.003 | 0.072 ± 0.002 | 0.066 ± 0.000 | 0.073 ± 0.001 | 0.072 ± 0.001 | 0.072 ± 0.001 | 0.066 ± 0.000 |
| Start-up interval | 24.538 ± 0.297 | 28.016 ± 0.094 | 27.192 ± 0.075 | 28.407 ± 0.228 | 28.444 ± 0.216 | 28.648 ± 0.503 | 27.334 ± 0.192 |
| Unattributed (within simulator clock) | 11.013 ± 0.601 | 11.125 ± 0.899 | 12.164 ± 0.882 | 11.079 ± 0.707 | 10.303 ± 1.147 | 11.044 ± 2.082 | 11.292 ± 2.297 |
| **Total wall time** | **1241.948 ± 2.311** | **1333.704 ± 2.756** | **1334.684 ± 1.067** | **311.729 ± 0.448** | **237.067 ± 1.676** | **213.901 ± 2.005** | **213.439 ± 2.441** |

**Supplementary Table S3.** Complete per-phase timing breakdown on the benchmark system. Mean ± standard deviation over n = 5 repetitions; per-phase values are those reported by the master MPI rank (rank 0), on which data merging and output writing are performed. The rows are exhaustive and sum to the total wall time: ‘Start-up interval’ is total wall time minus the master rank’s internally reported wall time — process launch, interpreter start-up on all ranks, GPU context creation and model-database opening — and ‘Unattributed’ is the remainder of the simulator’s own clock that is not assigned to a named phase.

| **Configuration** | **Metric** | **Rep 0 (warm-up)** | **Rep 1** | **Rep 2** | **Rep 3** | **Rep 4** | **Rep 5** |
| --- | --- | --- | --- | --- | --- | --- | --- |
| Container, CPU, 20 ranks | Total wall time | 1238.33 | 1245.13 | 1240.99 | 1239.24 | 1243.38 | 1241.01 |
|  | Solver (psolve) | 1103.88 | 1111.20 | 1106.00 | 1104.99 | 1109.14 | 1107.89 |
| Container, CPU, 18 ranks | Total wall time | 1383.88 | 1338.31 | 1332.33 | 1331.68 | 1334.22 | 1331.99 |
|  | Solver (psolve) | 1238.14 | 1192.52 | 1186.36 | 1186.39 | 1188.11 | 1186.51 |
| Native, CPU, 18 ranks | Total wall time | 1432.49 | 1335.99 | 1334.79 | 1333.78 | 1335.40 | 1333.46 |
|  | Solver (psolve) | 1284.89 | 1188.79 | 1187.44 | 1186.82 | 1188.29 | 1185.70 |
| Container, GPU, 18 ranks, 1 GPU | Total wall time | 311.98 | 311.35 | 312.25 | 311.60 | 311.30 | 312.16 |
|  | Solver (psolve) | 165.74 | 165.63 | 165.43 | 165.69 | 165.40 | 165.49 |
| Container, GPU, 18 ranks, 2 GPUs | Total wall time | 237.47 | 234.12 | 237.60 | 238.00 | 238.21 | 237.40 |
|  | Solver (psolve) | 91.70 | 91.61 | 91.74 | 91.70 | 91.57 | 91.61 |
| Container, GPU, 18 ranks, 3 GPUs | Total wall time | 212.20 | 214.06 | 210.45 | 215.54 | 214.95 | 214.52 |
|  | Solver (psolve) | 69.23 | 68.43 | 68.50 | 68.30 | 68.30 | 68.08 |
| Native, GPU, 18 ranks, 3 GPUs | Total wall time | 215.38 | 215.14 | 215.02 | 215.35 | 211.65 | 210.03 |
|  | Solver (psolve) | 68.25 | 67.55 | 67.73 | 67.65 | 67.79 | 67.47 |

**Supplementary Table S4.** Per-repetition raw timings on the benchmark system, in seconds, including the warm-up repetition that is excluded from all analyses.

| **Configuration A** | **Configuration B** | **Identical** | **Spikes** |
| --- | --- | --- | --- |
| Container CPU, 18 ranks | Container GPU, 1 GPU | **yes** | 2,992,658 |
| Container CPU, 18 ranks | Container GPU, 2 GPUs | **yes** | 2,992,658 |
| Container CPU, 18 ranks | Container GPU, 3 GPUs | **yes** | 2,992,658 |
| Container CPU, 18 ranks | Native CPU, 18 ranks | **yes** | 2,992,658 |
| Container CPU, 18 ranks | Native GPU, 3 GPUs | **yes** | 2,992,658 |
| Container GPU, 1 GPU | Container GPU, 2 GPUs | **yes** | 2,992,658 |
| Container GPU, 1 GPU | Container GPU, 3 GPUs | **yes** | 2,992,658 |
| Container GPU, 1 GPU | Native CPU, 18 ranks | **yes** | 2,992,658 |
| Container GPU, 1 GPU | Native GPU, 3 GPUs | **yes** | 2,992,658 |
| Container GPU, 2 GPUs | Container GPU, 3 GPUs | **yes** | 2,992,658 |
| Container GPU, 2 GPUs | Native CPU, 18 ranks | **yes** | 2,992,658 |
| Container GPU, 2 GPUs | Native GPU, 3 GPUs | **yes** | 2,992,658 |
| Container GPU, 3 GPUs | Native CPU, 18 ranks | **yes** | 2,992,658 |
| Container GPU, 3 GPUs | Native GPU, 3 GPUs | **yes** | 2,992,658 |
| Native CPU, 18 ranks | Native GPU, 3 GPUs | **yes** | 2,992,658 |

**Supplementary Table S5**. Numerical reproducibility on the benchmark system: all pairwise comparisons of the recorded spike output across execution arms and GPU counts (18 MPI ranks throughout). All 15 comparisons matched exactly, with identical spike times, identical global identifiers and 2,992,658 spikes in every configuration.

| **Metric** | **18 ranks (s)** | **20 ranks (s)** | **Difference, 20 − 18** |
| --- | --- | --- | --- |
| Solver (psolve) | 1187.98 | 1107.85 | −80.13 s (−6.75%) 95% CI [−81.74, −78.52] s |
| Total wall time | 1333.70 | 1241.95 | −91.76 s (−6.88%) 95% CI [−93.01, −90.50] s |

**Supplementary Table S6**. Justification of the CPU baseline used in Experiment 2: 20 MPI ranks versus 18 MPI ranks, containerized CPU arm (paired, n = 5). The 20-rank configuration uses every physical core of the benchmark machine and is also the faster of the two, so adopting it as the baseline does not flatter the GPU comparison; the 18-rank baseline would give the larger factors of 17.4× and 6.24×, which are also reported in Table S7. The equivalence of the containerized and native arms (Table 2 of the main text) was verified at 18 ranks; no native 20-rank run was performed, and we take that equivalence to hold at 20 ranks because the container cost arises in filesystem access and process launch and is independent of rank count.

| **Target configuration** | **Baseline configuration** | **Solver only** | **End-to-end** | **Scope ratio** |
| --- | --- | --- | --- | --- |
| Container, GPU, 18 ranks, 3 GPUs | Container, CPU, 20 ranks | 16.21× | 5.81× | 2.79× |
| Container, GPU, 18 ranks, 3 GPUs | Container, CPU, 18 ranks | 17.39× | 6.24× | 2.79× |
| Container, GPU, 18 ranks, 2 GPUs | Container, GPU, 18 ranks, 1 GPU | 1.81× | 1.31× | 1.37× |
| Container, GPU, 18 ranks, 3 GPUs | Container, GPU, 18 ranks, 1 GPU | 2.42× | 1.46× | 1.66× |

**Supplementary Table S7**

Experiment 2 — GPU acceleration. Speed-up factors within the containerized arm, each with its baseline and its scope stated explicitly (benchmark system, n = 5). Every ratio is container-versus-container; neither numerator nor denominator is a native run. ‘Solver only’ compares the psolve phase and ‘end-to-end’ compares total wall time; the two differ because only the solver is GPU-accelerated.

| **Configuration** | **Cell creation** | **Synapse creation** | **Connection creation** | **Cell parameter application** |
| --- | --- | --- | --- | --- |
| Container, CPU, 20 ranks | 23.79–24.81 s (4.2%) | 58.46–59.60 s (1.9%) | 2.77–2.97 s (7.2%) | 0.49–0.51 s (2.8%) |
| Container, CPU, 18 ranks | 25.74–26.72 s (3.7%) | 63.99–65.08 s (1.7%) | 2.99–3.22 s (7.6%) | 0.48–0.49 s (1.2%) |
| Native, CPU, 18 ranks | 26.04–26.80 s (2.9%) | 65.14–66.01 s (1.3%) | 3.00–3.13 s (4.5%) | 0.49–0.50 s (1.6%) |
| Container, GPU, 18 ranks, 1 GPU | 25.70–27.05 s (5.2%) | 63.61–65.45 s (2.8%) | 2.99–3.29 s (9.8%) | 0.48–0.49 s (1.6%) |
| Container, GPU, 18 ranks, 2 GPUs | 25.69–26.43 s (2.8%) | 63.78–64.65 s (1.4%) | 2.99–3.13 s (4.4%) | 0.48–0.49 s (1.0%) |
| Container, GPU, 18 ranks, 3 GPUs | 25.72–26.84 s (4.3%) | 63.78–65.31 s (2.4%) | 2.98–3.20 s (7.2%) | 0.48–0.49 s (1.4%) |
| Native, GPU, 18 ranks, 3 GPUs | 26.04–26.74 s (2.7%) | 64.78–65.86 s (1.7%) | 3.01–3.17 s (5.3%) | 0.49–0.50 s (2.3%) |

**Supplementary Table S8.** Across-rank variation of the model-construction phases on the benchmark system. Each entry gives the minimum and maximum, over MPI ranks, of that rank’s mean across the n = 5 repetitions, with the spread (max − min) / mean in parentheses. Model construction is the only stage with appreciable rank-to-rank imbalance. Its two largest components vary by up to 5.2% (cell creation) and 2.8% (synapse creation) between ranks; connection creation varies by up to 9.8% but accounts for only 3.0 s of the 96.1 s spent on construction. Solver time is identical across all ranks within a run, since the ranks synchronize at every spike-exchange interval.

| **Phase (s)** | **Container GPU, 1** | **Container GPU, 2** | **Container GPU, 3** |
| --- | --- | --- | --- |
| Cell creation | 60.682 ± 0.440 | 60.704 ± 0.409 | 60.825 ± 0.550 |
| Synapse creation | 157.936 ± 1.362 | 158.851 ± 1.810 | 158.703 ± 1.940 |
| Connection creation | 6.222 ± 0.138 | 6.158 ± 0.050 | 6.158 ± 0.070 |
| Cell parameter application | 0.378 ± 0.005 | 0.378 ± 0.005 | 0.380 ± 0.007 |
| Spike recording setup | 9.996 ± 0.178 | 9.866 ± 0.133 | 10.018 ± 0.103 |
| Voltage recording setup | 0.213 ± 0.001 | 0.212 ± 0.002 | 0.212 ± 0.002 |
| **Simulation (psolve)** | **414.056 ± 0.552** | **223.169 ± 0.563** | **161.595 ± 0.476** |
| Data gathering (alltoall) | 4.349 ± 0.061 | 4.331 ± 0.048 | 4.338 ± 0.031 |
| Data merging | 4.693 ± 0.138 | 4.715 ± 0.160 | 5.271 ± 0.571 |
| Output save | 0.046 ± 0.000 | 0.046 ± 0.001 | 0.048 ± 0.002 |
| Start-up interval | 208.277 ± 2.127 | 210.134 ± 2.288 | 207.815 ± 2.963 |
| Unattributed (within simulator clock) | 8.532 ± 1.457 | 8.264 ± 1.536 | 7.875 ± 1.843 |
| **Total wall time** | **875.379 ± 2.607** | **686.828 ± 2.935** | **623.237 ± 2.563** |

**Supplementary Table S9**

Complete per-phase timing breakdown on the portability system, using the same container image (6 MPI ranks). Mean ± SD over n = 5 repetitions; conventions are those of Table S3. These values are reported for completeness of the deposited record and are not comparable with those of the benchmark system, which differs in CPU and GPU generation and was run with a different number of MPI ranks.

| **Configuration** | **Metric** | **Rep 0 (warm-up)** | **Rep 1** | **Rep 2** | **Rep 3** | **Rep 4** | **Rep 5** |
| --- | --- | --- | --- | --- | --- | --- | --- |
| Container, GPU, 6 ranks, 1 GPU | Total wall time | 874.70 | 872.72 | 873.40 | 874.52 | 877.70 | 878.55 |
|  | Solver (psolve) | 413.35 | 413.87 | 414.67 | 414.09 | 413.23 | 414.41 |
| Container, GPU, 6 ranks, 2 GPUs | Total wall time | 686.55 | 685.51 | 689.32 | 690.60 | 684.31 | 684.40 |
|  | Solver (psolve) | 222.79 | 222.42 | 223.98 | 223.11 | 223.32 | 223.01 |
| Container, GPU, 6 ranks, 3 GPUs | Total wall time | 622.76 | 626.08 | 619.71 | 622.51 | 622.49 | 625.39 |
|  | Solver (psolve) | 157.90 | 160.94 | 161.92 | 162.05 | 161.81 | 161.26 |

**Supplementary Table S10**

Per-repetition raw timings on the portability system, in seconds, including the warm-up repetition. Layout and conventions are those of Table S4. Unlike the benchmark system, this matrix shows no warm-up penalty: the first repetition of each configuration falls within the spread of the five that follow. The first repetition is nonetheless discarded here as well, so that both systems are analyzed identically. These timings are reported for completeness of the deposited record and are not compared with those of the benchmark system.

| **System A** | **Configuration A** | **System B** | **Configuration B** | **Identical** | **Spikes** |
| --- | --- | --- | --- | --- | --- |
| Zeke | Container GPU, 1 GPU | Zeke | Container GPU, 2 GPUs | **yes** | 2,992,658 |
| Zeke | Container GPU, 1 GPU | Zeke | Container GPU, 3 GPUs | **yes** | 2,992,658 |
| Zeke | Container GPU, 2 GPUs | Zeke | Container GPU, 3 GPUs | **yes** | 2,992,658 |
| Eren | Container, 18 ranks, 3 GPUs | Zeke | Container, 6 ranks, 1 GPU | **yes** | 2,992,658 |
| Eren | Container, 18 ranks, 3 GPUs | Zeke | Container, 6 ranks, 2 GPUs | **yes** | 2,992,658 |
| Eren | Container, 18 ranks, 3 GPUs | Zeke | Container, 6 ranks, 3 GPUs | **yes** | 2,992,658 |

**Supplementary Table S11**

Numerical reproducibility on the portability system, and between the two systems. The same container image, copied unmodified from the benchmark system, was run with 6 MPI ranks to match the six physical cores of the portability machine. The upper block compares the three GPU-count configurations on that machine with one another; the lower block compares each of them with the benchmark system. Every comparison is exact, and the spike count is the same throughout, although the two systems differ in CPU generation, in GPU generation and in the number of MPI ranks. No timing claim is made across the two machines.
